# Evaluating the effects of consolidation on intrusion and retraction using temporary anchorage devices—a FEM study

**DOI:** 10.1186/s40510-016-0155-8

**Published:** 2017-01-09

**Authors:** Monica Namburi, Sleevaraju Nagothu, Chetan S. Kumar, N. Chakrapani, C. H. Hanumantharao, Supradeep K. Kumar

**Affiliations:** St. Joseph Dental College, Eluru, Andhra Pradesh India

**Keywords:** FEM, Mini-screws, Consolidation, Bilateral implants, Three implants, Intrusion, Retraction, PDL, Stress distribution, Initial displacements

## Abstract

**Background:**

Extraction of premolars and retracting the anterior teeth using mini-implants and anterior retraction hooks became advent now a day. In such treatments, consolidation of arches is not done in regular practice. So, the present study is concentrated on effects of consolidation in two implant and three implant combinations of retraction and intrusion.

**Methods:**

A three-dimensional FEM model of maxillary teeth and periodontal ligament housed in the alveolar bone with the first premolars extracted is generated with appropriate number of elements and nodes. The models were broadly divided into two groups according to the no. of implants. Mini-implants were placed bilaterally between the second premolar and molar at varying heights (7, 10, 13 mm) in group I, and along with bilateral implants, an additional mid-implant is placed between the central incisors as group II. Brackets with 0.022 slot were placed on the teeth, 19 × 25 SS wire is placed in the brackets, an anterior retraction hook was placed at 9 mm height, and analysis was done to evaluate the stresses and displacement patterns in consolidation and non-consolidation models.

**Results:**

The results showed that consolidation of the anterior teeth during intrusion and retraction shows various advantages such as less stresses on the bone, PDL, implant, teeth, and no labial flaring of the anterior teeth and three implant system, i.e., two bilateral implant at 10 mm and a mid-implant at 12 mm between the centrals has shown to be better than other models as bodily movement is observed.

**Conclusion:**

Consolidation is better than non consolidation during enmasse retraction and intrusion.

## Background

Dental protrusion is common in many ethnic groups around the world. It is characterized by dento-alveolar flaring of only maxillary teeth or both the maxillary and the mandibular anterior teeth with resultant protrusion of the lips and the convexity of the face [1]. Extracting the first four premolars and retracting the anterior segments with maximum anchorage is one of the ways to reduce the protrusion and to straighten the patient’s profile. The retraction of four incisors after canine retraction is accepted as a method to minimize the mesial movement of the posterior teeth segment, whereas en-masse retraction of six anterior teeth may create anchorage problems. Anchorage may be aided by the use of intra- and extra-oral appliances. However, intraoral anchorage devices may provide insufficient anchorage, whereas extra-oral appliances provide a sufficient anchorage but are dependent on patient compliance [2]. The goal of orthodontic treatment is to achieve the desired tooth movement with minimum unwanted side effects and to improve patient aesthetics [3].

In retraction mechanics, temporary anchorage devices (TADs) or orthodontic mini-implants (OMIs) are used for anchorage purposes [4, 5], which gained popularity and are used successfully in orthodontic treatment. Hooks are used on the archwire as force application points to achieve anterior retraction. These hooks or power arms can be crimped, screwed on, soldered to the archwire with silver solder, or welded [6]. The force is applied directly from the implant to the power arms with the help of e-chains or coil springs for effective space closure. This force vector can be controlled by changing mini-implant insertion height and/or anterior retraction hook height, thereby raising a number of different force action line alternatives. Orthodontists therefore, prior to mini-implant installation, should define which force action lines will be employed and determine the force vector which will exert upon the anterior teeth. In en-masse retraction consolidation of the arches which means making them as a single unit using ligature wire as the anterior (canine to canine) and posterior segments (premolar to molars on one side as one segment and on the other side as another segment) should be done. Consolidation of arches is advised to inhibit the unwanted tooth movement.

Application of engineering knowledge in dentistry with the use of computational techniques has helped to understand oral biomechanics aspects. One of such software is finite element analysis (FEM) [7]. This method can simplify the physiologic responses of dento-alveolar complex to orthodontic forces by exhibiting quantitative data, and is recently preferred by the researchers of the field [8]. The main advantage of using finite element analysis is that many alternative designs can be tried out for their validity, safety, and integrity using the computer, even before the first prototype is built [9]. In spite of the significant advances that have been made in developing finite element models, the results obtained must be carefully examined before they can be used.

Many studies evaluated the stress in the bone [10, 11], PDL [12–18], implants [19–22] in mini-implant-assisted retraction [23] with different height of implants and retraction hooks [24, 25] and materials [26, 27]. But there are no studies which evaluated the stresses on the bone and PDL in two implant and three implant combinations. There are no studies regarding the effects of consolidation during retraction. So, the present study was conducted to evaluate the analysis of stresses and displacement pattern in two implant and three implant combinations with and without consolidation.

## Methods

**Fig. 1:**
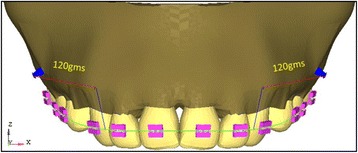
Bilateral implant model

In this study, 12 finite element models were created with 43,887 nodes and 209,807 elements (Tables 1 and 2) with variations in number of mini-implants, height of placement of bilateral mini-implants, and different force levels from the mid-implant and with and without consolidation. The FEM model used in the present study is having the first premolars extracted, and the implants were placed between the roots of the second premolars and first molars [6, 28] to retract the anterior teeth. The present study includes the implant of width 1.3 mm × 8 mm [28–30]. Bilateral implants will be angulated to 30° to the long axis of the occlusal plane. The anterior retraction hook is placed between the canine and lateral brackets [31] at a 9-mm height [30, 32], (Fig. 1). The PDL space is maintained equal at 0.25 mm in all teeth and isotropic linear properties are given for PDL [1, 33, 34].

**Table 1:** Material properties

Material	Modulus of elasticity (MPa)	Poisson’s ratio
Tooth	80,000	0.3
PDL	0.1	0.45
Stainless steel wire and brackets	210 × 10^3^	0.3
Cortical/hard bone	13,800	0.26
Trabecular/soft bone	345	0.31
Titanium implants	110 × 10^3^	0.3

**Table 2:** Nodes and elements

S.No.	Description	Number of elements	Number of nodes
1	Hard bone	79,455	25,150
2	Soft bone	94,844	24,957
3	Mini-implants	14,259	4470
4	Brackets	52,730	16,856
5	Wire	100	101
6	Teeth	20	22
7	PDL	46,570	13,640
8	Anterior retraction hook	20	22

Total nodes in the study = 43,887. Total elements in the study = 209,807

**Fig. 2:**
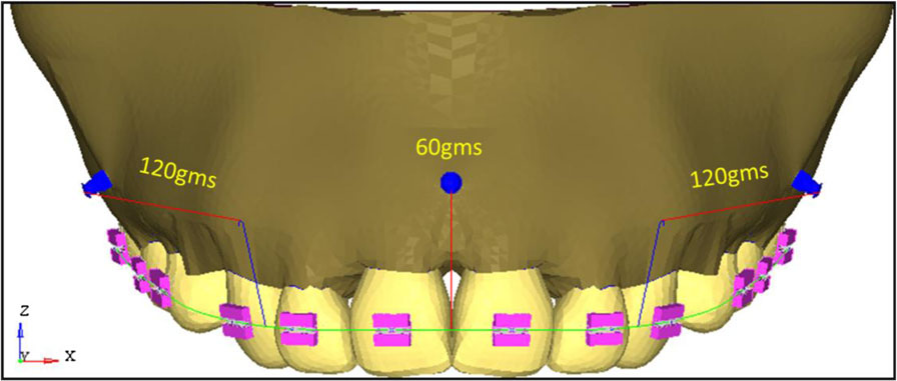
Three implant model

**Fig. 3:**
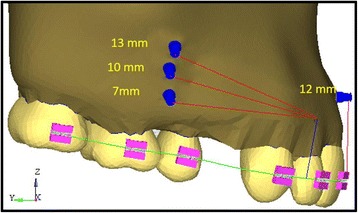
Implant heights

The mid-implant is placed perpendicular to the occlusal plane. The anterior retraction hook is placed constant at 9 mm from the archwire oriented gingivally and a force of 120 g was given from bilateral implants for retraction and a force of 60 g is given from mid-implant for intrusion purpose (Fig. 2). The stress distribution patterns in teeth, PDL, and bone along with anterio-posterior and vertical displacements are observed in consolidation, non-consolidation in two implant and three implant systems at low pull (7 mm), medium pull (10 mm), and high pull (13 mm) bilateral implant heights from the arch wire and in three implant models the mid-implant is placed constant at 12-mm height from the archwire (Fig. 3). The models were broadly divided into two groups.

- Group I: Bilateral implants placed at different heights (7, 10, and 13 mm) in between the second premolar and first molar.
- Group II: Along with bilateral implants (7, 10, and 13 mm), an additional mid-implant is placed in between the two central incisors at 12-mm height from the arch wire.

**Fig. 4:**
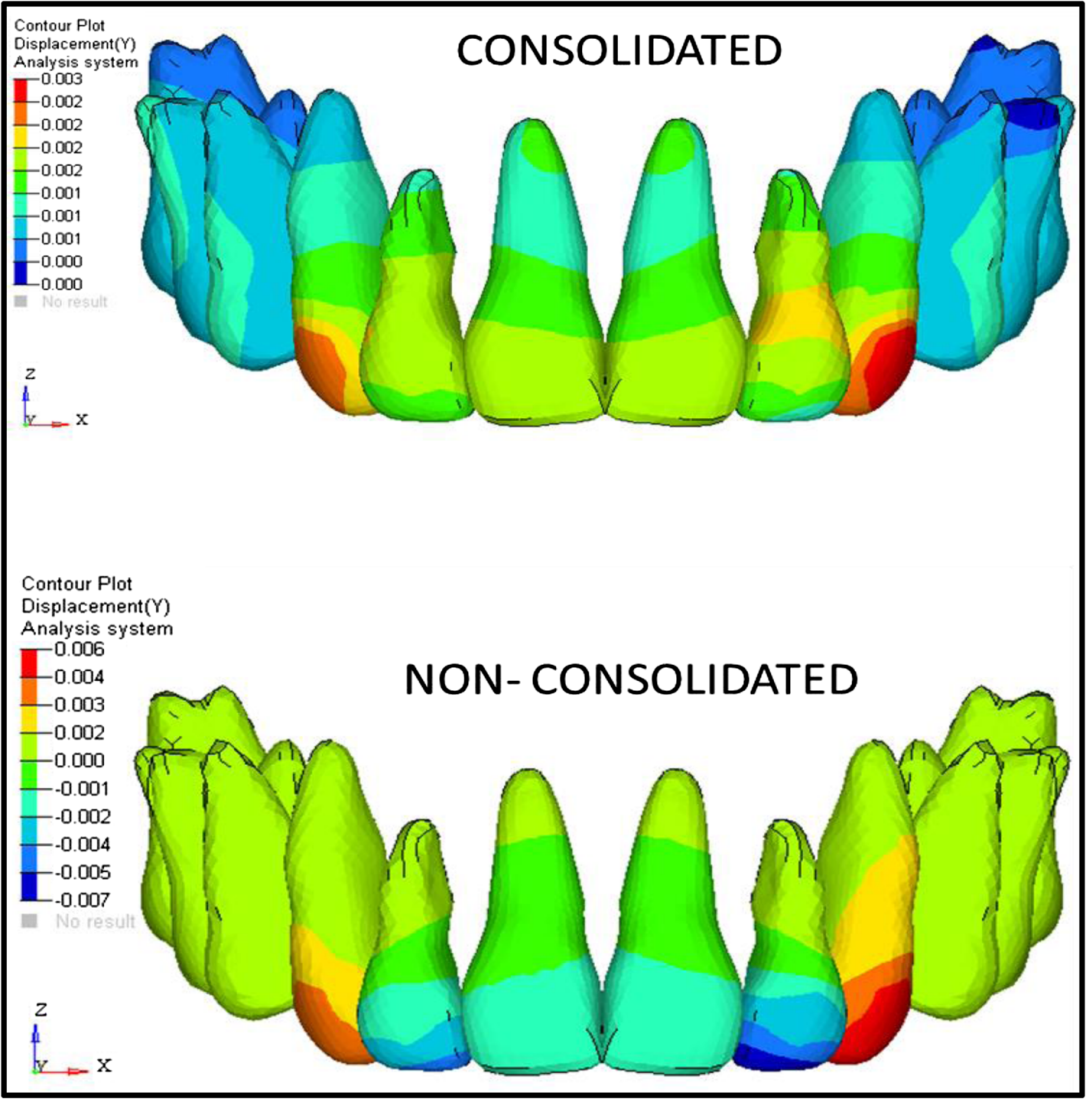
Anterio-posterior displacement pattern

**Fig. 5:**
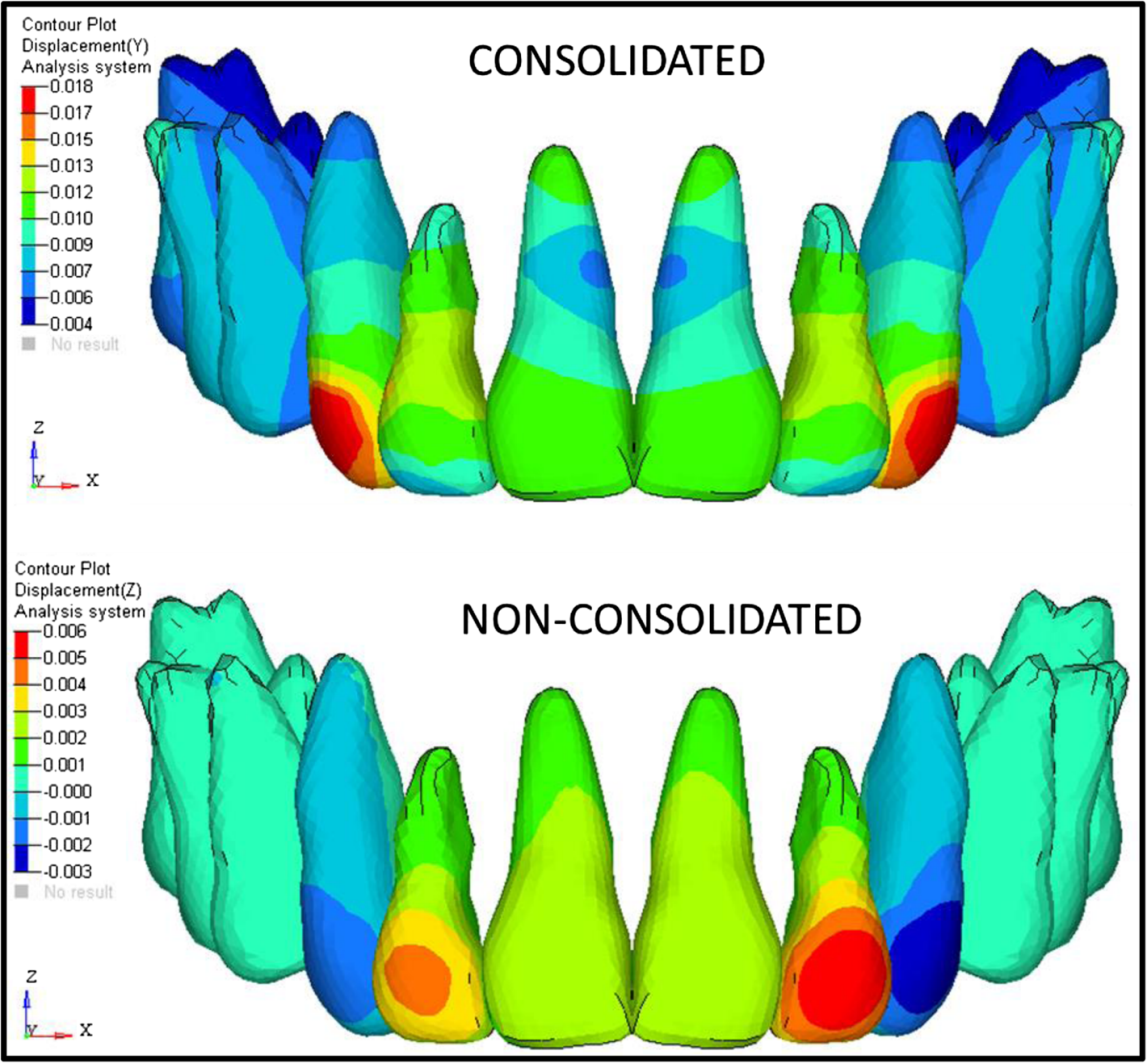
Vertical displacement patterns

**Fig 6:**
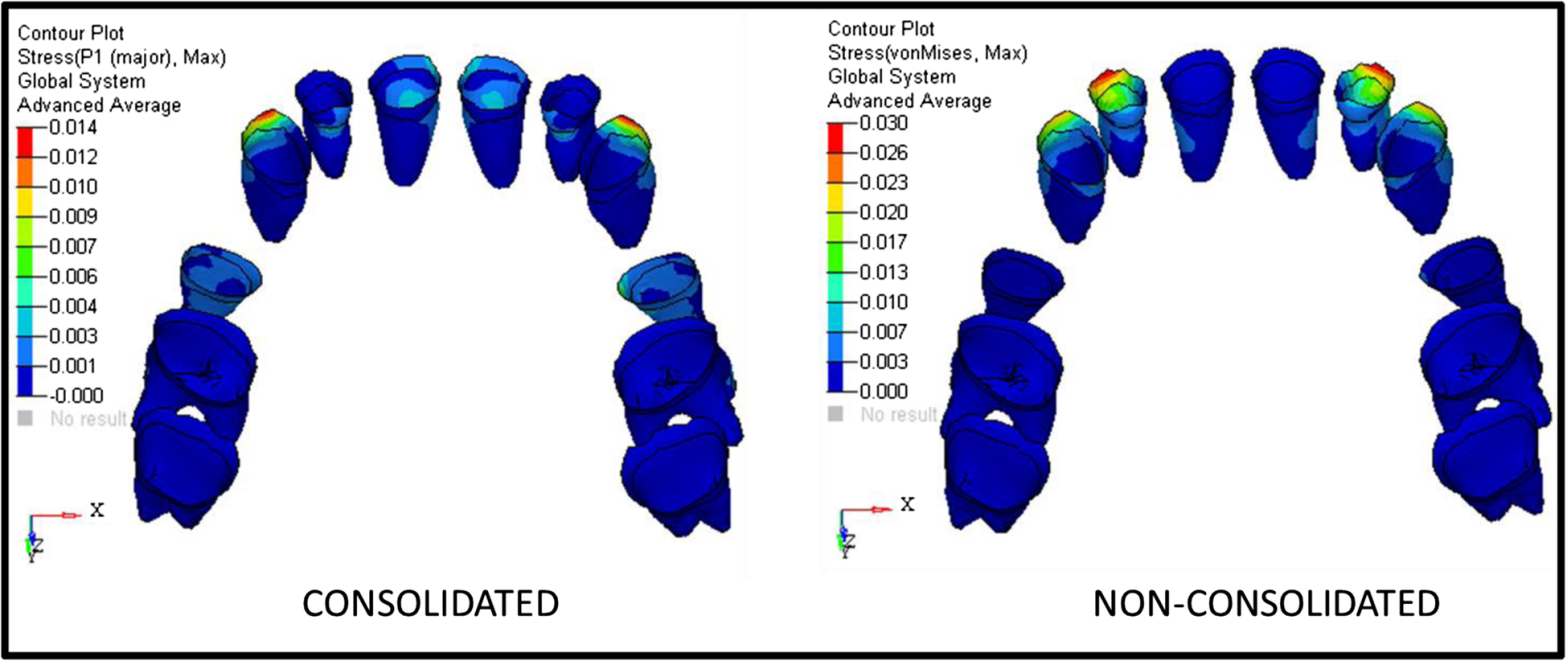
Stress distribution patterns in PDL

**Fig. 7:**
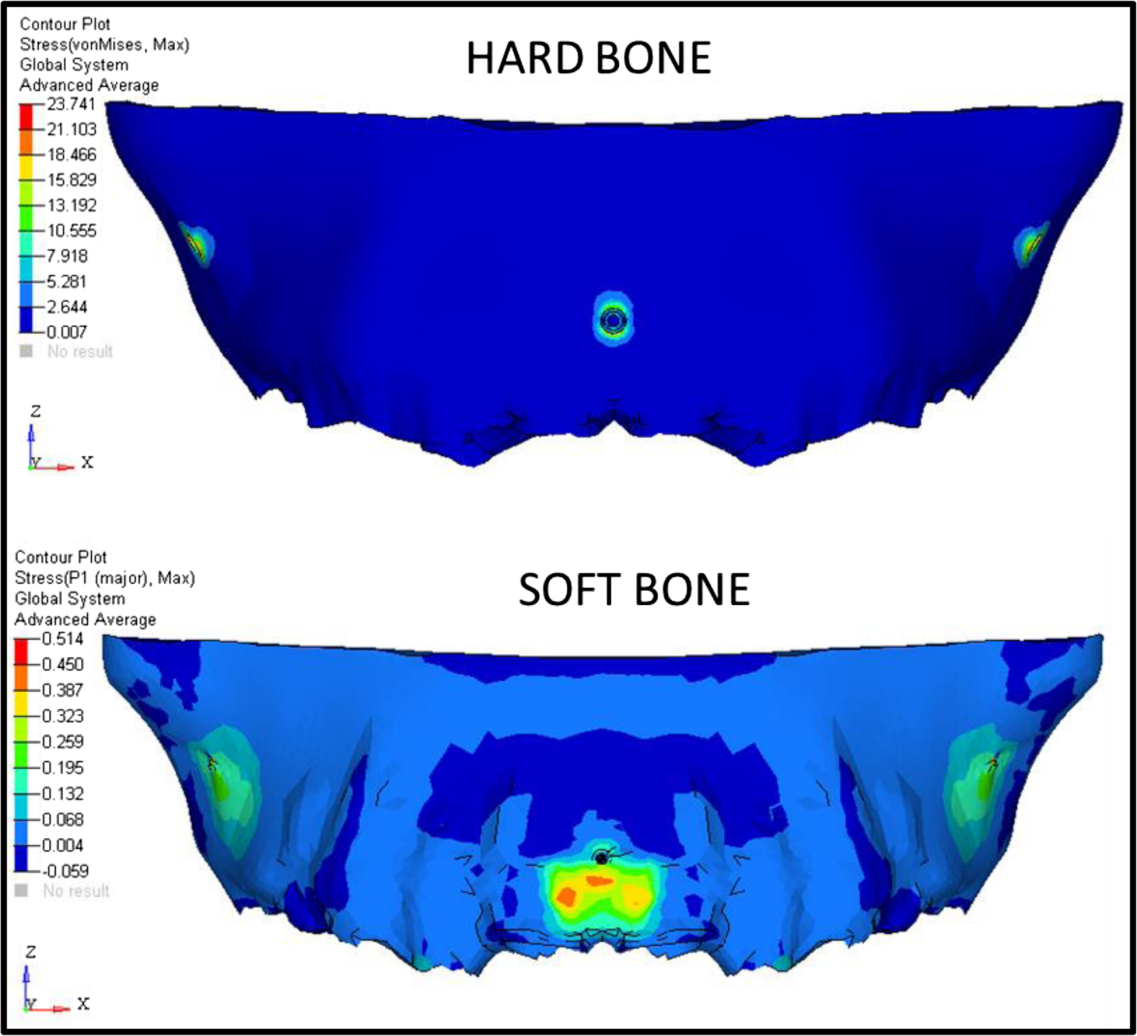
Stress distribution patterns in the hard bone and soft bone

**Fig. 8:**
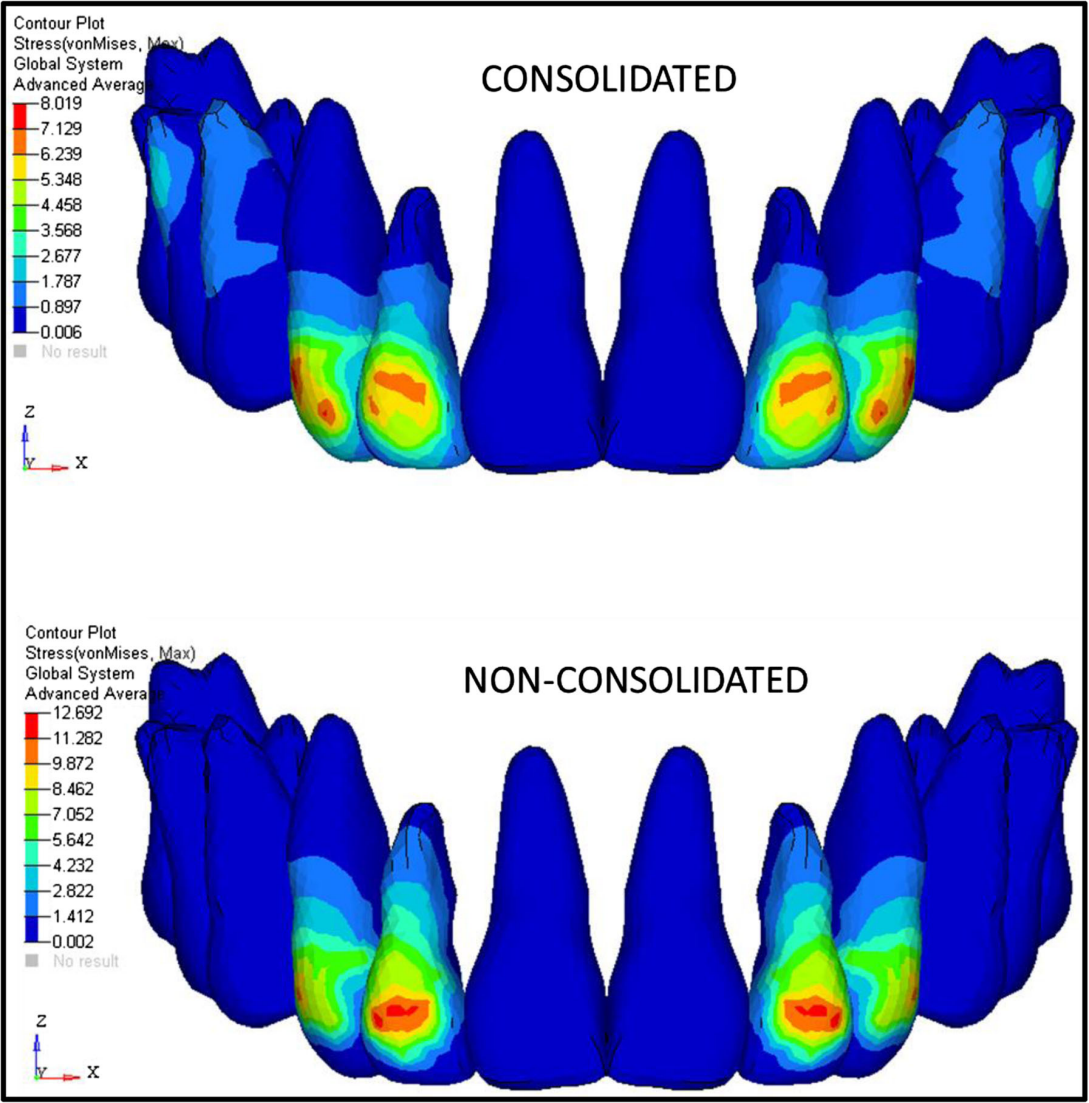
Stress distribution patterns on the teeth

The analysis was carried out using ANSYS 12.1 version software in Bangalore, INDIA. The initial displacement patterns in anterio-posterior direction (Fig. 4, Table 3), vertical direction (Fig. 5, Table 4), and stress patterns in the PDL (Fig. 6, Table 5), bone (Fig. 7, Table 6), teeth (Fig. 8, Table 7), and implant in two implant and three implant scenarios at different implant heights were analyzed (Table 8).

**Table 3:** Anterio-posterior displacements in consolidated and non-consolidated model in group I and group II models (mm)

	Implant heights (mm)	Central incisor				Lateral incisor				Canine			
		Consolidated		Non-consolidated		Consolidated		Non-consolidated		Consolidated		Non-consolidated	
		Crown	Apex	Crown	Apex	Crown	Apex	Crown	Apex	Crown	Apex	Crown	Apex
Group I	7	0.027	0.012	0.013	0.01	0.023	0.017	−0.022	0.021	0.033	0.011	0.054	0.011
	10	0.024	0.011	0.01	0.009	0.022	0.015	−0.029	0.018	0.03	0.009	0.049	0.01
	13	0.021	0.009	0.005	0.008	0.017	0.013	−0.034	0.015	0.026	0.008	0.045	0.008
Group II	7	0.01	0.0125	−0.011	0.011	0.009	0.01	−0.013	0.01	0.013	0.009	0.021	0.009
	10	0.002	0.001	−0.002	0.001	0.001	0.001	−0.003	0.002	0.002	0.001	0.006	0.001
	13	0.012	0.011	−0.022	0.009	0.008	0.009	−0.049	0.009	0.017	0.006	0.034	0.006

Inference (positive(+) : palatal movement; negative(-): labial movement)

Group I: In consolidation, palatal tipping is seen, and in non-consolidation, labial flaring is seen in lateral incisor

Group II: In consolidation, 10-mm implant height model showed bodily movement, 7- and 13-mm implant height showed palatal tipping and in non-consolidation, labial flaring of the teeth observed in centrals and laterals

**Table 4:** Vertical displacements in group I and group II consolidated and non-consolidated models (mm)

		Consolidated			Non-consolidated		
		Central	Lateral	Canine	Central	Lateral	Canine
Group I	7 mm	−0.006	0.007	−0.016	−0.002	0.0027	−0.026
	10 mm	−0.004	−0.004	−0.01	0	−0.02	−0.026
	13 mm	−0.003	0.012	−0.014	0.005	0.035	−0.023
Group II	7 mm	0.006	0.007	−0.003	0.016	0.016	−0.009
	10 mm	0.001	0.002	−0.002	0.002	0.006	−0.003
	13 mm	0.008	0.019	−0.008	0.023	0.044	−0.02

Inference: (positive (+) : intrusion; negetive(-): extrusion)

Group I: In consolidation, extrusion of canines and centrals observed in all cases, laterals extruded in 10-mm implant height and in other models intrusion of laterals observed. In non-consolidation, extrusion of canines observed in all cases whereas laterals showed extrusion in 10-mm implant height and centrals showed extrusion in 7-mm implant height, intrusion of centrals and laterals observed in 13-mm implant height model

Group II: In consolidation and non-consolidation, extrusion of canines observed and centrals and laterals intruded in all models

**Table 5:** Stresses on PDL in Mega Pascal in group I and group II in consolidation models

		Von Mises stresses					Principle tensile stresses					Principle compressive stresses				
	Implant height (mm)	C.I	L.I	C	PM	M	C.I	L.I	C	PM	M	C.I	L.I	C	PM	M
Group I	7	0.004	0.016	0.011	0.004	0.004	0.003	0.003	0.013	0.005	0.003	0	0	0	0	0
	10	0.002	0.017	0.013	0.004	0.004	0.004	0.003	0.013	0.003	0.003	0	0	0	0	0
	13	0.002	0.017	0.011	0.002	0.002	0.004	0.003	0.013	0.003	0.003	0	0	0	0	0
Group II	7	0.002	0.003	0.002	0.002	0.009	0.003	0	0	0.011	0.001	0	0	0	0	0
	10	0.004	0.017	0.013	0.004	0.002	0.004	0.003	0.014	0.003	0.002	0	0	0	0	0
	13	0.004	0.017	0.013	0.003	0.002	0.004	0.003	0.013	0.003	0	0	0	0	0	0

Inference: *C.I* central incisor, *L.I* lateral incisor, *C* canine, *PM* premolar, *M* molar

Group I: Highest von Mises stress are observed in laterals (0.017) at 10- and 13-mm implant height followed by canines at 10-mm implant height (0.013) and centrals at 7-mm implant height (0.004). Principle tensile stresses are more in canines in all models (0.013) and no compressive stresses registered

Group II: Principle tensile stresses are high in premolars in 7-mm implant height model (0.011) and von Mises stresses are more in 10- and 13-mm implant height models in laterals (0.017) and canines (0.013). Principle tensile stresses are more in canines in 10- and 13-mm implant height models (0.014 and 0.013, respectively), and no compressive stresses are observed

**Table 6:** Stresses on PDL in group I and group II non-consolidated models (MPa)

		Von Mises stresses					Principle tensile stresses					Principle compressive stresses				
	Implant heights (mm)	C.I	L.I	C	PM	M	C.I	L.I	C	PM	M	C.I	L.I	C	PM	M
Group I	7	0.003	0.028	0.019	0.003	0.003	0	0.005	0.026	0.002	0.002	0.001	0	−0.001	−0.001	−0.001
	10	0.003	0.03	0.023	0.004	0.004	0.006	0.009	0.028	0.002	0.002	0.001	−0.001	−0.001	−0.001	−0.001
	13	0.007	0.03	0.023	0.002	0.002	0.006	0.006	0.027	0.003	0.003	0.001	−0.001	−0.001	0	0
Group II	7	0.003	0.01	0	0.003	0.007	0.003	0.003	0.011	0.003	0.003	0	0	0	0	0
	10	0	0.03	0.026	0	0	0.006	0.006	0.028	0	0	0.001	−0.001	−0.001	−0.001	0
	13	0.003	0.03	0.027	0.003	0.002	0.006	0.006	0.027	0.002	0.003	0.001	−0.001	−0.001	−0.001	0

Inference: +ve: tensile stresses; –ve: compressive stresses

Group I: Von Mises stresses are more in laterals in 10- and 13-mm implant heights (0.030) in canines at 10- and 13-mm implant heights (0.023) and centrals in 13-mm implant height (0.007). Principle tensile stresses are more in 10-mm canines (0.028) and laterals (0.009) and centrals (0.006) at 10- and 13-mm implant heights. Compressive stresses are present in all teeth except in 7-mm implant height in lateral, 13-mm implant height in premolars and molars

Group II: Von Mises stresses are more in canines in 10- and 13-mm implant heights (0.026 and 0.027, respectively). Principle tensile stresses are more in canines in 10- and 13-mm implant height (0.028 and 0.027, respectively). Compressive stresses are not observed in 7-mm implant height, and stresses are not observed in molars at 10- and 13-mm implant heights

**Table 7:** Stresses on the hard bone and soft bone around the implant area in consolidated and non-consolidated in group I and group II models (Mpa)

		Max von Mises stress in consolidation		Max von Mises stress in non-consolidation		Max principal stress in consolidation		Max principal stress in non-consolidation	
		Hard	Soft	Hard	Soft	Hard	Soft	Hard	Soft
Group I	7 mm	29.04	0.99	29.1	0.99	32.85	1.15	32.9	1.15
	10 mm	25.9	0.6	26	0.6	29.3	0.72	29.3	0.72
	13 mm	22.35	0.5	22.42	0.5	26.17	0.56	26.26	0.56
Group II	7 mm	35.9	1.24	35.9	1.24	40.9	1.39	41	1.39
	10 mm	25.67	0.6	25.75	0.6	29.35	0.7	29.45	0.7
	13 mm	23.74	0.049	23.59	0.5	31.04	0.51	30.85	0.51

Group I: Von Mises stresses and principle stresses are more in both consolidations and non-consolidation in the hard and soft bone at 7-mm implant height

Group II: Von Mises stresses and principle stresses are more in both consolidations and non-consolidation in the hard and soft bone at 7-mm implant height

**Table 8:** Results

	Consolidated	Non-consolidated
Anterio-posterior	Palatal tipping	Labial flaring
Vertical	Less intrusive force	More intrusive force
Stress on PDL	Less stress	More stress
Stress on the hard bone	Less stress	More stress
Stress on the teeth	Less stress	More stress

## Discussion

The goal of the present study is to compare and evaluate the effects of consolidation on intrusion and retraction in two implant and three implant scenarios.

In this study, 12 finite element models were created with variations in number of mini-implants, height of placement of bilateral mini-implants and different force levels from the mid-implant and with and without consolidation.

The bilateral implants will be angulated to 30° to the long axis of the occlusal plane as per the studies conducted by Kyung HM, Park HS et al. [2], Ju-Eun Lim [35], and Fathima Jasmine [36] where they stated that if the mini-implant is angulated, the cortical bone and implant interface area will be increased as the most of the implant will be in the cortical bone, and thus, the stability will be increased and also the proper angle of insertion is important for cortical anchorage, patient’s safety, and biomechanical control and mid-implant is placed perpendicular to the occlusal plane. According to Madhupura et al. [28] and Masahiro Iijima [30], the implant of 8 mm × 1.3 mm is placed because longer miniscrew implants were associated with a greater incidence of sinus and bi-cortical perforations. According to Tina Chugh et al. [37], the highest cortical bone density was observed between the second premolar and first molar. So, the implant was placed at the second premolar and first molar regions. In Gjessing’s [30] rule of thumb, the position of the center of resistance of the anterior segment will be at 9–10 mm apically, so the anterior retraction hook is placed constant at 9 mm from the archwire oriented gingivally and a force of 120 g was given from bilateral implants for retraction and a force of 60 g is given from mid-implant for intrusion (Figs. 1 and 2). The stress distribution patterns in teeth, PDL, and bone along with anterio-posterior and vertical displacements are observed in consolidation, non-consolidation in two implant and three implant systems at low pull (7 mm), medium pull (10 mm), and high pull (13 mm) bilateral (Fig. 3) implant heights from the archwire, and in three implant models, the mid-implant is placed constant at 12-mm height from the archwire. The models were broadly divided into two groups.

- Group I: Bilateral implants placed at different heights (7, 10, and 13 mm) in between the second premolar and first molar (Fig. 1).
- Group II: Along with bilateral implants (7, 10, and 13 mm), an additional mid-implant is placed in between the two central incisors at 12-mm height from the archwire (Fig. 2).

Comparing the consolidation and non-consolidation, the consolidated arches in group I showed more palatal tipping whereas non-consolidated models showed labial flaring of the teeth as crown moves labially and apex moves palatally, which is unfavorable. Whereas in group II, bodily movements are observed in consolidation and in non-consolidation system, the labial flaring of centrals and laterals are observed. The center of resistance for the present study model might be located at 10-mm height as the bodily movement is seen in 10-mm implant and 9-mm anterior retraction hook with consolidation in group II. The force levels passing away from the center of resistance will cause tipping and at the center of resistance causes bodily movement which is proved correct in the present study. Abhishek Parashar et al. [38] conducted a study and inferred that the bodily movement with very minimal torque loss was observed with 8-mm implant and concluded the same.

In vertical displacements, it is concluded that intrusion is seen in three implant system as expected. Intrusive movement was more in the high pull implant than in the low pull and medium pull implant in all cases. The force levels passing from the high pull implant to the retraction hook will cause intrusion and from the medium pull implants shows bodily movement and low pull implant showed tipping forces. A study conducted by Shrinivas S Ashekar et al. [39] suggest that the force levels passing from the high pull implant to the retraction hook will cause intrusion and from the medium pull implant shows bodily movement and the low pull implant showed tipping forces which supports the present study. So, the high pull implants show high intrusive effect, and the canines showed extrusion in all cases as the retraction force is more towards the canine as the tipping of the canines is observed. In consolidation and non-consolidation systems, the intrusion effect is high in non-consolidated system than in the consolidated system as the force are distributed among the teeth in consolidated arches, so less intrusive effect is seen in consolidation.

The present study showed highest stresses on lateral incisors, and this may be due to the short roots of the lateral incisors [40]. Burstone and Viecilli [41] stated that it is a natural concept that larger teeth have more PDL and root support than smaller teeth, and hence, when the same load is applied, the stress magnitudes in the PDL for larger teeth are smaller and larger for smaller teeth. Consequently, the resistance to tooth movement of larger teeth is larger compared to smaller teeth. The maxillary molars suggested that widely divergent roots will require higher loads (causing rotation about the vertical axis of the root) to achieve similar levels of stress, even if the surface area of the root is similar to other less divergent tooth roots and this supports the present study [42].

Stresses on PDL are high in laterals and canines compared to other teeth in all cases, and in 7 mm with mid-implant model (group II), the stresses are more in the posterior teeth than in the anterior teeth and consolidated system showed less stresses when compared with the non-consolidated system. Unfavorable compressive stresses are seen in non-consolidated group, which concluded that consolidation is better than non-consolidation as the stresses are distributed in consolidation system.

Stresses on the hard bone at the implant area in consolidation and non-consolidation in group I and group II inferred that the stresses on the implant area are more in non-consolidated models than in consolidated models in all groups, and the von Mises stresses are equal in group II 7-mm implant model. Stresses on the soft bone around the implant area in consolidation and non-consolidation at different implant heights are equal in all models. In 13-mm three implant model (group II), von Mises stresses are more in non-consolidated model than in consolidated models. Seven-millimeter implants showed highest stresses in the hard and soft bone in all cases. This concludes that low pull implants with high anterior hook produces more stresses in the bone around the implant area (Table 9).

**Table 9:** Stresses in the implant in group I and group II consolidated and non-consolidated models

		Max von Mises stress in consolidation	Max von Mises stress in non-consolidation	Max principal stress in consolidation	Max principal stress in non-consolidation
Group I	7 mm	108.4	108.7	116	116.2
	10 mm	106.6	106.9	116.2	116.5
	13 mm	106.1	106.4	109.5	109.8
Group II	7 mm	135.1	135.1	144.5	144.5
	10 mm	106.6	106.9	116.2	116.6
	13 mm	106.1	106.4	109.5	109.8

Group I: Von Mises stresses are high in 7-mm implant height, and principle stresses are more in 10-mm implant height in consolidation and non-consolidation

Group II: Von Mises stresses and principle stresses are high in 7-mm implant height in consolidation and non-consolidation

Stresses on the teeth showed more stresses on laterals. This is supported by the study conducted by S. Reimann [30] as the study showed the similar results. But in group II, 7-mm implant height model showed highest stresses on the posterior teeth. In consolidation and non-consolidation in group I and group II, it is inferred that the stresses are more in the non-consolidated models than in the consolidated models in all cases. This suggests that consolidation is important in the case of en-masse retraction with anterior retraction hook (Table 10).

**Table 10:** Stresses on teeth (laterals) in group I and group II consolidated and non-consolidated models

		Max von Mises stress in consolidation	Max von Mises stress in non-consolidation	Max principal stress in consolidation	Max principal stress in non-consolidation
Group I	7 mm	8.02	11.26	6.04	9.45
	10 mm	8.4	12.4	6.4	10.5
	13 mm	8.11	12.6	6.17	10.73
Group II	7 mm	3.24	4.92	2.92	3.98
	10 mm	8	12.85	6.23	10.93
	13 mm	8.15	13.1	5.9	11.15

Inference: (laterals recorded the highest stresses in all models)

Group I: In consolidation, 10-mm implant height and non-consolidation 13-mm implant height showed highest von Mises stresses. Principle tensile stresses are high in 10-mm consolidation implant model and 13-mm non consolidation implant model

Group II: In consolidation and non-consolidation, von Mises stresses are high in 13-mm implant height, and principle stresses are more in 10-mm in consolidation and 13-mm in non-consolidation implant heights

So, the present study concludes that consolidation is better than non-consolidation in retraction using implant with anterior retraction hook, and intrusion is effective in three implants than bilateral implants.

## Conclusions

The following important conclusions are drawn from this study:

1. Bodily movement was observed when implant was placed at 10-mm height in both group I and group II, this seems to be the ideal height for bilateral implant position for retraction of the anterior teeth.
2. In three implant system, more intrusion is seen than two implant system. The three implant system is better for intrusion of the anterior teeth.
3. The stresses in PDL are more in the two implant system than in the three implant system in all the anterior teeth.
4. Undesirable labial flaring of teeth are observed in non-consolidation in two implant and three implant system.
5. The stresses on the hard bone, PDL, and implant showed less stresses in consolidation and more in non-consolidation.
6. Intrusion and retraction is better in consolidated arches and also less stresses were observed on the teeth, PDL, bone, and implant.
7. Force levels passing from the high pull implant to the retraction hook will cause intrusion and from the medium pull implant shows bodily movement and low pull implant showed tipping forces in consolidated arches.

This study suggests that with consolidation of the anterior teeth, desirable tooth movement can be achieved with less stresses on all surrounding structures.
